# Comparison of combined spinal-epidural versus general anesthesia with epidural catheter on postoperative quality of recovery after abdominal hysterectomy: a prospective observational study

**DOI:** 10.1186/s12871-025-03252-2

**Published:** 2025-07-31

**Authors:** Mehmet G. Taflan, Sevda Akdeniz, Hatice Kusderci, Kübra Arslan, Mural Ünal, Mustafa Süren, Serkan Tulgar

**Affiliations:** 1 Department of Anesthesiology and Reanimation, Samsun University Samsun Training and Research Hospital, Kışla, Barış Avenue, No:199, Ilkadım, Samsun, 55090 Türkiye; 2 Bahçeşehir University, Göztepe Medical Park Hospital, Department of Anesthesiology and Reanimation, Istanbul, Türkiye

**Keywords:** Hysterectomy, Spinal anesthesia, Epidural analgesia, Quality of recovery (QoR), Patient-controlled analgesia, Postoperative nausea and vomiting (PONV)

## Abstract

**Background:**

We aimed to evaluate the effect of combined spinal-epidural (CSE) anesthesia versus general anesthesia with an epidural catheter (GE) on the quality of postoperative recovery in abdominal hysterectomy patients. The recovery outcomes were assessed using the Quality of Recovery-15 (QoR-15) scale.

**Methods:**

This prospective, single-center observational study included 87 female patients (aged 18–75 years). Their ASA physical status varied from I to III, and they had a planned elective abdominal hysterectomy scheduled. We divided them into two groups based on the type of anesthesia administered: the CSE group and the GE group. The primary outcome consisted of the total QoR-15 score we measured 24 h after the operation. Among the secondary outcomes were the incidence of postoperative nausea and vomiting (PONV), analgesic consumption, pain scores assessed using the Numerical Rating Scale (NRS), the need for rescue analgesia, time to mobilization, hospitalization duration and the surgeon satisfaction score.

**Results:**

At 24 h post-surgery, the CSE group had a significantly higher QoR-15 score compared to that of the GE group, with scores of 131.97 ± 8.67 and 122.93 ± 13.41, respectively (*p* = 0.001). Additionally, the CSE group required less analgesic consumption, averaging 119.53 ± 33.16 ml compared to 149.32 ± 53.11 ml in the GE group (*p* = 0.002). The need for rescue analgesia was also lower in the CSE group, with 9.30% of patients requiring it compared to 27.27% in the GE group (*p* = 0.031). Furthermore, pain scores measured using the NRS and the PONV incidence were significantly lower in the CSE group during the first three hours after surgery (*p* < 0.001). However, there were no significant differences in the time to mobilization, length of hospital stays, or surgeon satisfaction scores between both groups.

**Conclusion:**

Combined spinal-epidural anesthesia provides a better quality of postoperative recovery for patients undergoing abdominal hysterectomy. This technique improves pain control, reduces the need for opioids, and minimizes nausea and vomiting. These findings suggest that combined spinal-epidural anesthesia may enhance patient comfort and well-being during recovery.

**Supplementary Information:**

The online version contains supplementary material available at 10.1186/s12871-025-03252-2.

## Background

Hysterectomy ranks among the most carried out gynecological surgeries in women [1]. While hysterectomy can address various gynecological issues, it may lead to certain physical and psychological effects. In addition to its role as a reproductive organ, the uterus plays a part in other physiological processes and is often seen as a symbol of youth and sexuality [2]. Consequently, undergoing a hysterectomy may bring about complicated psychological effects, such as depression, anxiety and guilt [3]. Beyond psychological outcomes, hysterectomy patients may face physical issues, such as pain, nausea, and vomiting as a result of the surgical method used. Depending on the clinical situation, a hysterectomy can be performed using minimally invasive techniques or more invasive approaches, such as the abdominal method [4, 5]. Abdominal hysterectomy presents challenges in managing postoperative pain. Inadequate pain control can lead to delayed mobilization, prolonged hospital stays, increased complication rates and decreased patient satisfaction [6].

Several quality recovery scales have been developed to assess the diverse postoperative outcomes from the patient’s perspective. The QoR-15 is one of these tools. It holistically evaluates psychological, physiological and functional aspects, particularly notable for its simplicity and quick administration [7]. Due to its features, the QoR-15 scale has become widely used for assessing how perioperative anesthetic and analgesic interventions affect recovery quality.

Abdominal hysterectomy can be performed using general or neuraxial anesthesia techniques. An epidural catheter is often recommended to control postoperative pain [8]. Although there is extensive literature on how different postoperative analgesia regimens affect pain management and analgesic consumption, relatively few recent studies utilize the quality of recovery scale to explore how intraoperative anesthesia techniques affect the quality of postoperative recovery [9–11]. Specifically, the impact of combined anesthetic techniques on postoperative recovery, as assessed by a comprehensive tool like the QoR-15, has not been thoroughly investigated. Further research is needed to assess the impact of various anesthetic techniques on the recovery quality following the abdominal hysterectomy, especially using the QoR-15 scale. The objective of the present study is to evaluate how the postoperative recovery quality differs between patients receiving combined spinal-epidural (CSE) and those undergoing general anesthesia paired with an epidural catheter (GE).

## Methods

### Study design

The present study was an observational, prospective, single-center, parallel-cohort design study approved by the Samsun University Clinical Research Ethics Committee (Decision No: 2024/6/7) and conducted following the updated 2013 Declaration of Helsinki [12]. It was registered on ClinicalTrials.gov (registration number: NCT06461832) before enrolling participants. Patient recruitment occurred at Samsun Education and Research Hospital from April 2024 to February 2025. All participants gave written informed consent, permitting the use and publication of their data. Throughout the study, participant privacy and data security were strictly maintained. All personal information was securely stored and accessible only to authorized research team members.

### Participants

We included female patients (18 to 75 years old) with an American Society of Anesthesiologists (ASA) physical status of I to III. They were scheduled to undergo an elective abdominal hysterectomy. Exclusion criteria included emergency surgery, any contraindications to neuraxial anesthesia, malignancies outside the uterus, dependence on alcohol or substances, illiteracy, or refusal to participate. The study followed the guidelines of the Strengthening the Reporting of Observational Studies in Epidemiology (STROBE) [13].

### Anesthesia techniques and procedures

All patients received one of two standard protocols used in our department: GE or CSE anesthesia. The study excluded patients who required alternative anesthesia techniques. During the preoperative visit, participants were informed about the QoR-15 scale and how the patient-controlled analgesia (PCA) device would be used. One hour before surgery (T0), each participant completed the QoR-15 questionnaire without any intervention from the researchers. Baseline QoR-15 scores were collected to ensure comparability between groups, as previous research has shown that poorer preoperative QoR-15 scores are associated with worse postoperative recovery outcomes [14]. The anesthesiologist and the surgeon, who were not part of the study, determined the anesthesia method applied to participants based on their daily clinical practice. To minimize bias, both the investigators and outcome assessors were blinded to the assigned anesthesia technique.

*In the CSE group (n = 43)*, patients were administered intravenous sedation using midazolam at a dosage of 0.03 mg/kg. Subsequently, they underwent spinal anesthesia with a 25G Quincke spinal needle at the L3–L4 or L4–L5 intervertebral level with 15 mg of 0.5% hyperbaric bupivacaine. An epidural catheter (Egemen COMBİFİX^®^ Combined Spinal – Epidural Anesthesia Set) was inserted through the same intervertebral space utilizing the loss-of-resistance technique and was advanced four cm within the epidural space, once the spinal block was established.

*In the GE Group (n = 44)*, patients received intravenous sedation with midazolam at a dosage of 0.03 mg/kg. Using the loss of resistance technique, an epidural catheter was then inserted at the L3–L4 or L4–L5 intervertebral levels, advancing it four cm into the epidural space. General anesthesia was initiated with propofol, dosed at 2 mg/kg, with muscle relaxation achieved through rocuronium at 0.6 mg/kg and thereafter endotracheal intubation. Anesthesia was maintained using 2% sevoflurane and remifentanil at a rate ranging from 0.1 to 0.25 mcg/kg/min. At the end of the procedure, neuromuscular blockade was reversed using atropine and neostigmine. Strict aseptic techniques and sterile skin preparation were utilized in both groups to reduce the risk of infection.

### Analgesic management during the perioperative period

During the surgical procedure, all participants received standard care, which included intravenous acetaminophen (1 g), dexketoprofen (50 mg), and ondansetron (8 mg) for pain management and antiemetic prophylaxis. After the surgery, a PCA device (BD BodyGuard Color Vision™) was connected to the epidural catheter. The PCA solution was formulated with 1 mg/ml of bupivacaine and 2 mcg/ml of fentanyl, totaling 250 ml. A 10 ml bolus was set to be delivered, with a 30-minute lockout period between doses and a maximum dose of 100 ml over four hours. This epidural PCA regimen reflects our institutional protocol and is based on clinical practices supported by existing literature. The combination of bupivacaine and fentanyl, as well as the programmed intermittent bolus technique, has been shown to provide effective analgesia with favorable safety and recovery profiles in various perioperative settings [15–17]. No medications were administered through the epidural catheter during the surgery; the consumption of PCA solution in the first 24 h was recorded to compare analgesic requirements between the groups.

### Postoperative management

Upon completion of the surgery, all patients were moved to the recovery unit and were monitored every 15 min during the first hour for pain as well as postoperative nausea and vomiting (PONV). We evaluated pain with an 11-point Numerical Rating Scale (NRS: 0–10). In cases of an NRS score of four or higher, rescue analgesia was administered intravenously at 0.5 mg/kg tramadol. PONV was evaluated with a 5-point verbal descriptive scale, where 0 represented no symptoms, 1 indicated mild nausea, 2 meant severe nausea, 3 referred to a single episode of vomiting, and 4 corresponded to multiple episodes of vomiting. Patients with a PONV score more excellent than 2 received 0.15 mg/kg metoclopramide intravenously. When the patients’ modified Aldrete score reached 9 or higher, they were transferred from the recovery unit to the ward. In the ward, they were given 1 g of acetaminophen three times daily (every eight hours). If the NRS score remained 4 or higher despite receiving epidural PCA, 0.5 mg/kg tramadol was administered intravenously as rescue analgesia.

### Outcomes

The total QoR-15 score 24 h post-surgery (T1) was the main outcome. The QoR-15 includes 15 items that assess recovery from different aspects: pain, physical independence, psychological support, physical comfort, and emotional state. Each item is scored on a scale from 0 (never) to 10 (always). The total score varied from 0 to 150, with the former signifying inadequate recovery quality, and the latter indicating excellent quality of recovery. Based on the total score, the quality of recovery is categorized into four groups: poor (0–89), moderate (90–121), and excellent (136–150) [18]. The QoR-15 instrument’s reliability and validity have been established including its Turkish version. We selected the QoR-15 due to its strong psychometric properties, including high reliability, validity, and sensitivity to clinical changes. Its concise structure enables rapid completion while maintaining measurement accuracy, offering a practical advantage over the longer QoR-40, particularly in high-demand clinical settings where minimizing patient burden and optimizing time efficiency are essential [7, 19]. At time point T1, a blinded investigator assisted participants in completing the questionnaire without influencing their responses.

The secondary outcomes measured in this study included the total volume of medication administered through the PCA device during the postoperative 24 h, the number of those who needed rescue analgesics or antiemetics, the PONV score, any intraoperative complications, the time taken for mobilization, the duration of hospitalization, and the surgeon satisfaction level.

Time to mobilization was defined as the number of hours from the patient’s admission to the ward until they could transfer independently to a chair. The length of hospital stay was calculated as the number of days from the postoperative ward admission until discharge. Surgeon satisfaction regarding intraoperative conditions was assessed using a uni-dimensional 0–10 numeric rating scale, where 0 indicated the worst and 10 indicated the best surgical experience. This single-item score was used to evaluate overall surgical relaxation and maneuverability, which are closely interrelated and frequently assessed together in clinical studies. This evaluation was performed immediately after the completion of the procedure to ensure accurate and real-time feedback.

### Sample size calculation

Data from an earlier study [20] that reported QoR-15 global scores were used to determine the sample size: the treatment group had a score of 110.44 ± 21.12, while the control group scored 94.51 ± 16.19 based on an effect size of 0.846568, an alpha level of 0.05 (α), a power rate of 95% (1-β), and statistical power analysis with G*Power (version 3.1.9.6) revealed that a minimum of 38 patients per group (76 in total) was required. To account for potential dropouts, we aimed to enroll at least 42 patients in each group.

#### Statistical analysis

Statistical analyses were conducted using IBM SPSS Statistics (Version 23 for Windows, IBM Corp., Armonk, NY), and a *p*-value below 0.05 was considered to indicate statistical significance. The Shapiro–Wilk test was applied to evaluate the normality of data distribution. Depending on the characteristics of the data, categorical variables were analyzed using Pearson’s chi-square test, or Fisher’s exact test with Monte Carlo simulation when appropriate. Pairwise comparisons were assessed using the Bonferroni-adjusted z-test. The continuous variables were then compared between the groups using the independent samples t-test for normally distributed data and the Mann–Whitney U test for non-normally distributed data. Spearman correlation analysis was performed to assess the relationship between patient-reported outcomes and clinical discharge time. Categorical variables are presented as frequencies and percentages, whereas continuous variables are reported as mean ± standard deviation or as median with interquartile ranges (Q1–Q3).

## Results

**Fig. 1:**
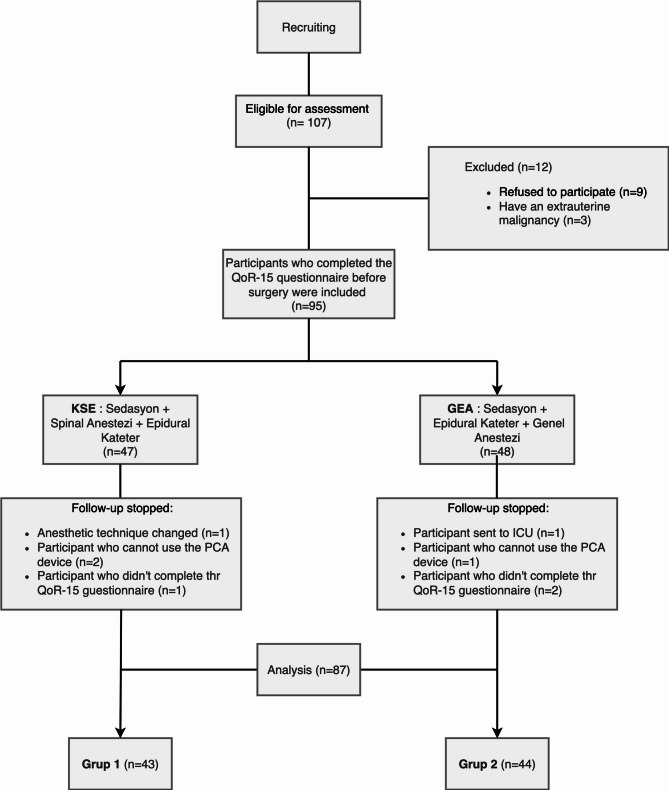
STROBE flow diagram of patient enrollment, exclusion, and final group allocation. CSE: Combined spinal epidural, GE: General Anesthesia with an Epidural Catheter, PCA: Patient Control Analgesia, ICU: Intensive Care Unit

We initially evaluated 107 patients for eligibility. Twelve were excluded from the study: nine refused participation and three had extrauterine malignancies. Of the remaining 95 patients, eight were lost to follow-up— four from each of the CSE and GE groups. Forty-three patients from the CSE group and 44 patients from the GE group, 87 patients in total, completed the study (see Fig. 1).

The two groups had comparable baseline characteristics and intraoperative variables. We found no statistically significant differences regarding age, weight, height, ASA status, preoperative QoR-15 scores, or intraoperative hypotension and bradycardia. These findings indicate a consistent distribution of initial conditions and intraoperative experiences across both groups (see Table 1).

**Table 1:** Demographic and clinical characteristics of patients in CSE and GE groups

	CSE (*n* = 43)	GE (*n* = 44)	*p*
	Mean ± SD Median [Q1-Q3]	Mean ± SD Median [Q1-Q3]	
Age (years)	48.02 ± 6.46 47 [44–50]	48.23 ± 9.16 47 [43–51.5]	0.905^*^
Weight (kg)	75.76 ± 11.07 75 [69–85]	74.11 ± 10.67 74 [67–81]	0.480^*^
Height (cm)	158.51 ± 4.64 159 [155–162]	158.25 ± 3.64 159.5 [155.5–160]	0.942^*^
ASA n, (%)			
I	6 (13,9)	10 (22,7)	0.564^**^
II	25 (58,1)	32 (72,7)	
III	2 (4,6)	2 (4,5)	
Preoperative QoR-15	143.95 ± 5.14 (142.41–145.49) [140.4–147.27]	139.95 ± 10.00 (137- 142.9) [133.5–146.71]	0.231^*^
Intraoperative Hypotension, n (%)	12 (27.90)	7 (15.90)	0.176^**^
Intraoperative Bradycardia, n (%)	5 (11.62)	1 (2.27)	0.085^**^

Data are presented as mean ± standard deviation (SD) with 95% confidence intervals (CI) and median with interquartile ranges [Q1–Q3] for continuous variables, and as number and percentage [n (%)] for categorical variables

Q1: 25th Percentile, Q3: 75th Percentile

*SD* Standard Deviation, *CI* Confidence Interval, *IQR* Interquartile Range, Values presented in bold indicate statistically significant differences at *p* < 0.05

^*^Comparisons of non-normally distributed continuous variables between the two groups were performed using the Mann-Whitney U test

^**^Categorical variables were analyzed using Pearson’s Chi-Square test**

At 24 h after surgery, the total QoR-15 score was higher in the CSE group than in the GE group, with 131.97 ± 8.67 versus 122.93 ± 13.41 (*p* = 0.001). A detailed analysis of the individual items revealed that the CSE group performed significantly better in several areas: “Able to return to work or usual home activities” (*p* = 0.042), “Feeling comfortable and in control” (*p* = 0.027), “Having a feeling of general well-being” (*p* = 0.027), “Nausea or vomiting” (*p* = 0.039), and “Feeling worried or anxious” (*p* = 0.018). In the remaining QoR-15 items, both groups demonstrated no significant differences (see Table 2).

**Table 2:** Comparison of postoperative QoR-15 total and item-specific scores between CSE and GE groups

	CSE (*n* = 43)	GE (*n* = 44)	*p*
	Mean ± SD (95% CI) Median [Q1-Q3]	Mean ± SD (95% CI) Median [Q1-Q3]	
Postoperative QoR-15 score	131.97 ± 8.67 (129.38–134.56) 132 [124–139]	122.93 ± 13.41 (118.97–134.56) 123 [112–134]	**0.001**
Able to breathe easily	9.81 ± 0.50 (9.66–9.96) 10 [10–10]	9.45 ± 1.02 (9.15–9.75) 10 [9–10]	0.167
Been able to enjoy food	9.41 ± 0.85 (9.16–9.66) 10 [9–10]	8.70 ± 1.71 (8.19–9.21) 9 [8–10]	0.115
Feeling rested	8.83 ± 1.42 (8.41–9.25) 9 [8–10]	8.36 ± 2.03 (7.76–8.96) 9 [7.5–10]	0.436
Have had a good sleep	8.60 ± 1.32 (8.21–8.99) 9 [7–10]	7.97 ± 2.07 (7.36–8.58) 9 [7–10]	0.283
Able to look after personal toilet and hygiene unaided	6.81 ± 2.61 (6.03–7.59) 7 [4–9]	5.38 ± 3.30 (4.4–6.36) 5 [2.5–8]	0.064
Able to communicate with family or friends	9.79 ± 0.55 (9.63–9.95) 10 [10–10]	9.63 ± 0.71 (9.42–9.84) 10 [9.5–10]	0.373
Getting support from hospital doctors and nurses	9.72 ± 0.66 (9.52–9.92) 10 [10–10]	9.38 ± 1.18 (9.03–9.73) 10 [9–10]	0.299
Able to return to work or usual home activities	7.02 ± 2.16 (6.37–7.67) 8 [5–9]	5.68 ± 3.00 (4.79–6.57) 5.5 [3–8]	**0.042**
Feeling comfortable and in control	9.04 ± 1.04 (8.73–9.35) 9 [8–10]	8.15 ± 1.85 (7.6–8.7) 9 [7–9]	**0.027**
Having a feeling of general well-being	9 ± 1.19 (8.64–9.36) 9 [9–10]	8.40 ± 1.45 (7.97–8.83) 9 [8–9]	**0.027**
Moderate pain	7.65 ± 1.41 (7.23–8.07) 8 [7–9]	7.34 ± 1.77 (6.82–7.86) 8 [6–8.5]	0.597
Severe pain	8.69 ± 1.79 (8.15–9.23) 9 [8–10]	8.95 ± 1.55 (8.49–9.41) 9 [9–10]	0.602
Nausea or vomiting	9.30 ± 1.10 (8.97–9.63) 10 [9–10]	8.45 ± 1.77 (7.93–8.97) 9 [7–10]	**0.039**
Feeling worried or anxious	8.93 ± 1.24 (8.56–9.3) 9 [9–10]	8.09 ± 1.80 (7.56–8.62) 8.5[7 − 9]	**0.018**
Feeling sad or depressed	9.30 ± 1.42 (8.88–9.72) 10 [9–10]	8.88 ± 1.64 (8.4–9.36) 9 [8–10]	0.067

Data are presented as mean ± standard deviation (SD) with 95% confidence intervals (CI) and median with interquartile ranges [Q1–Q3]. Postoperative QoR-15 total scores were compared between groups using the Independent Samples t-test, while item-specific scores were analyzed with the Mann-Whitney U test

Q1: 25th Percentile, Q3: 75th Percentile

*SD* Standard Deviation, *CI* Confidence Interval, *IQR* Interquartile Range, Values presented in bold indicate statistically significant differences at *p* < 0.05

The evaluation of the QoR-15 subdimensions revealed that the CSE group scored significantly higher than the GE group in two specific areas: “physical independence” (CSE: 13.84 ± 4.46 vs. GE: 11.07 ± 5.51; *p* = 0.023) and “emotional state” (CSE: 36.27 ± 3.20 vs. GE: 33.54 ± 4.70; *p* = 0.005). However, we found no significant differences in the other subdimensions, which included pain, physical comfort, and psychological support (see Table 3).

**Table 3:** Comparison of QoR-15 subdimension scores between CSE and GE groups

	CSE (*n* = 43)	GE (*n* = 44)	*p*
	Mean ± SD (95% CI) Median [Q1-Q3]	Mean ± SD (95% CI) Median [Q1-Q3]	
Pain	16.35 ± 2.87 (15.49–17.21) 17 (15–18)	16.30 ± 2.86 (15.45–17.15) 17 (15–18)	0.843
Physical Comfort	36.67 ± 3.30 (35.68–37.66) 37 (35–40)	34.50 ± 5.56 (32.86–36.14) 36 (31–39)	0.081
Physical Independence	13.84 ± 4.46 (12.51–15.17) 15 (10–18)	11.07 ± 5.51 (9.44–12.7) 10 (7–15)	**0.023**
Psychological Support	19.51 ± 1.12 (19.18–19.84) 20 (20–20)	19.02 ± 1.72 (18.51–19.53) 20 (19–20)	0.110
Emotional State	36.27 ± 3.20 (35.31–37.23) 37 (34–39)	33.54 ± 4.70 (32.15–34.93) 35 (30–37)	**0.005**

Data are presented as mean ± standard deviation with 95% confidence intervals (CI) and median [Q1–Q3]. Subdimension scores were compared between groups using the Mann-Whitney U test

Q1: Percentile 25; Q3: Perecntile 75

*SD* standard deviation, *CI* Confidence Interval, Values presented in bold indicate statistically significant differences at *p* < 0.05

In the age-based subgroup analysis, no statistically significant differences were observed between the CSE and GEA groups in patients aged 18–44 years regarding QoR-15 scores or any of its subdimensions (*p* > 0.05). In contrast, among patients aged 45–64 years, those who received CSE exhibited significantly higher QoR-15 scores compared to the GEA group (*p* < 0.001). Furthermore, this group also showed significantly greater scores in the physical comfort (*p* = 0.008), physical independence (*p* = 0.012), and emotional state (*p* = 0.012) subdimensions (see Table 4).

**Table 4:** Comparison of postoperative QoR-15 and subdimension scores between CSE and GEA groups in patients aged 18–44 and 45–64 years

	CSE (*n* = 43)	GE (*n* = 44)	*p*
	Mean ± SD (95% CI) Median [Q1-Q3]	Mean ± SD (95% CI) Median [Q1-Q3]	
QoR-15			
18–44	126.46 ± 8.39 (121.39−131.53)	119.67 ± 14.65 (111.55-127.78)	0.278
	125 [121–130]	117 [108–132]	
45–64	134.37 ± 7.76 (131.47-137.26)	123.85 ± 11.62 (119.15-128.54)	**< 0.001**
	134 [129–141]	123 [119–132]	
Pain			
18–44	15.46 ± 4.22 (12.91–18.01)	15.73 ± 2.74 (14.22–17.25)	0.780
	17 [14–18]	15 [14–18]	
45–64	16.73 ± 2.02 (15.98–17.49)	16.46 ± 2.85 (15.31–17.61)	0.946
	17 [15–18]	17 [16–18]	
Physical Comfort			
18–44	43.92 ± 4.15 (41.41–46.43)	42.13 ± 6.29 (38.65–45.62)	0.627
	44 [42–46]	43 [40–47]	
45–64	46.87 ± 2.83 (45.81–47.92)	43.15 ± 5.53 (40.92–45.39)	**0.008**
	47 [46–49]	44 [39–48]	
Physical Independence			
18–44	12.38 ± 4.41 (9.72–15.05)	9.80 ± 6.46 (6.22–13.38)	0.246
	11 [10–17]	9 [6–14]	
45–64	14.47 ± 4.41 (12.82–16.11)	11.27 ± 4.67 (9.38–13.16)	**0.012**
	16 [10–18]	11 [7–15]	
Psychological Support			
18–44	19.62 ± 1.12 (18.94–20.29)	19.00 ± 1.51 (18.16–19.84)	0.173
	20 [20–20]	20 [18–20]	
45–64	19.47 ± 1.14 (19.04–19.89)	19.12 ± 1.90 (18.35–19.88)	0.689
	20 [20–20]	20 [19–20]	
Emotional State			
18–44	35.08 ± 3.93 (32.70-37.45)	33.00 ± 4.97 (30.25–35.75)	0.286
	36 [34–37]	35 [30–36]	
45–64	36.80 ± 2.76 (35.77–37.83)	33.77 ± 4.71 (31.87–35.67)	**0.012**
	38 [35–39]	34 [31–37]	

Data are presented as mean ± standard deviation with 95% confidence intervals (CI) and median [Q1-Q3]. The Independent Samples t-test was used for normally distributed variables, and the Mann-Whitney U test was applied for non-normally distributed variables. A *p*-value < 0.05 was considered statistically significant. Group sizes: CSE 45–64 years (*n* = 30), GE 45–64 years (*n* = 26); CSE 18–44 years (*n* = 13), GEA 18–44 years (*n* = 15)

Q1: Percentile 25; Q3: Percentile 75

*SD* standard deviation, *CI* Confidence Interval, Values presented in bold indicate statistically significant differences at *p* < 0.05

According to the four-grade classification based on QoR-15 scores [11], the CSE group exhibited a significantly higher proportion of patients with “good” recovery (55.81%) compared to the GE group (34.09%). Additionally, a smaller percentage of patients had “moderate” recovery in the CSE group (9.30%) when compared to that of the GE group (43.18%) (*p*-value = 0.001. Notably, no patients in the CSE group experienced “poor” recovery, while 2.27% of patients in the GE group did (See Table 5 for details).

**Table 5:** Severity classification of postoperative recovery according to QoR-15 in CSE and GE groups

QoR-15 Categories *n*, (%)	CSE (*n* = 43)	GE (*n* = 44)	*p*
Excellent	15 (34.88)	9 (20.45)	0.001^*^
Good	24 (55.81)^b^	15 (34.09)^a^	
Moderate	4 (9.30)^b^	19 (43.18) ^a^	
Poor	0 (0.00)	1 (2.27)	

Values are presented as number and percentage [n (%)]. Postoperative recovery severity was categorized based on QoR-15 total scores: Excellent (≥ 135), Good (122–134), Moderate (90–121), Poor (< 90)

^*^Monte Carlo corrected Fisher’s Exact Test

^a, b^Groups sharing the same letter do not differ significantly, values presented in bold indicate statistically significant differences at p < 0.05.

Regarding the secondary outcomes, the CSE group had significantly lower PCA consumption during the first 24 h, with an average of 119.53 ± 33.16 ml compared to 149.32 ± 53.11 ml in the GE group (*p* = 0.002). Additionally, fewer patients in the CSE group required rescue analgesics, with rates of 9.30% versus 27.27% in the other group (*p* = 0.031). NRS scores were significantly reduced in the first hour postoperatively (measured at 0, 15, 30 and 60 min) (*p* < 0.001 for all time points) and this benefit continued for up to three hours (*p* = 0.013). Nevertheless, we noted no significant differences at the 6, 12, or 24-hour marks. The CSE group also had a significantly reduced PONV incidence (*p* < 0.001). Regarding antiemetic use, time to mobilization, length of hospital stays, or surgeon satisfaction, the groups showed no significant differences, as seen in Table 6.

**Table 6:** Comparison of secondary recovery outcomes between CSE and GE groups

	CSE (*n* = 43)	GE (*n* = 44)	*p*
	Mean ± SD (95% CI) Median [Q1-Q3]	Mean ± SD (95% CI) Median [Q1-Q3]	
PCA consumption in first 24 h, (ml)	119.53 ± 33.16 (109.62–29.44) 120 [100–140]	149.32 ± 53.11(133.63–165.01) 150 [110–180]	**0.002**
NRS;			
0th min	0.63 ± 1.07 0 [0–1]	3.77 ± 1.68 4 [3–5]	**< 0.001**
15th min	1.07 ± 1.26 1 [0–2]	4.02 ± 1.28 4 [3–5]	**< 0.001**
30th min	1.12 ± 1.16 1[0 − 2]	3.82 ± 1.17 4 [3–5]	**< 0.001**
60th min	1.4 ± 1.35 2 [0–2]	3.27 ± 1.32 3 [2–4]	**< 0.001**
3rd hr	2.23 ± 1.25 2 [1–3]	3.36 ± 2.18 3 [2–4]	**0.013**
6th hr	2.05 ± 1.25 2 [1–3]	2.64 ± 1.71 2.5 [1.5–3]	0.109
12th hr	1.86 ± 1.30 2 [1–3]	2.09 ± 1.03 2 [1–3]	0.284
24th hr	1.37 ± 1.48 1 [0–2]	1.48 ± 1.11 1 [1–2]	0.211
Patients given rescue analgesic in first 24 h, n (%)	4 (9.30)	12 (27.27)	**0.031**
Patients used antiemetic drug in first 24 h, n (%)	5 (11.62)	7 (15.90)	0.563
^*^PONV, n (%)			
0	36 (83.72)	13 (29.54)	**< 0.001**
1	1 (2.32)	13 (29.54)	
2	1 (2.32)	11 (25)	
3	4 (9.30)	4 (9.09)	
4	1 (2.32)	3 (6.81)	
Time to first mobilization (hr)	7,98 ± 0.94 (7.7–8.26) 8 [8–8]	9,24 ± 3.4 (8.24–10.24) 8 [8–8]	0.119
Time to discharge (day)	3.51 ± 0.91 (3.24–3.78) 3 [3–4]	3,91 ± 1.25 (3.54–4.28) 4 [3–5]	0.121
Surgeon satisfaction score	9.81 ± 0.5 (9.66–9.96) 10 [10–10]	9.84 ± 0.48 (9.7–9.98) 10 [10–10]	0.729

Continuous variables are expressed as mean ± SD (95% CI) and median [Q1–Q3]; categorical variables are given as number and percentage [n (%)]

Q1: Percentile 25; Q3: Perecntile 75

*SD* standard deviation, *CI* Confidence Interval, *NRS* Numerical Rating Scale, *PCA* patient-controlled analgesia, Values presented in bold indicate statistically significant differences at *p* < 0.05

^*^PONV: postoperative nausea and vomiting. PONV severity was categorized as: 0 = none, 1 = mild nausea, 2 = moderate nausea, 3 = single episode of vomiting, 4 = multiple episodes of vomiting. Comparisons were made using Mann–Whitney U or Chi-square/Fisher’s exact test as appropriate

Additionally, a Spearman correlation analysis between the QoR-15 item “Able to return to work or usual home activities” and actual discharge time showed no significant association (*r* = 0.068, *p* = 0.494), indicating that patient-perceived readiness for normal activities may not directly correspond to clinical discharge decisions, which are influenced by institutional routines and medical judgment.

## Discussion

Our findings in this observational study showed that CSE anesthesia improves postoperative recovery, as measured by the QoR-15 scale, compared to (GE) in patients undergoing abdominal hysterectomy.

The advantages of neuraxial techniques for postoperative recovery have been well-established for many years [21–23]. These techniques can significantly decrease the need for opioids after surgery, which in turn enhances patient comfort and satisfaction [24, 25]. Previous research has demonstrated a negative correlation between opioid consumption and the quality of recovery [10].

Studies comparing neuraxial and non-neuraxial techniques have shown significant differences in overall recovery scores and all related subdimensions [10, 26]. We found that global QoR-15 scores were significantly higher in the CSE group. However, only two subdomains - emotional state and physical independence - showed notable differences. CSE may provide more consistent pain relief by utilizing the initial analgesic effect of spinal anesthesia, which is maintained postoperatively through the epidural catheter. A study comparing CSE and epidural analgesia during labor suggested that CSE’s early-onset spinal block component significantly improves patient comfort and satisfaction [27]. Similarly, multiple studies have shown that spinal anesthesia is associated with lower early postoperative NRS scores than general anesthesia [28, 29]. When pain-induced stress and anxiety are alleviated, patients may experience enhanced emotional well-being. In line with this, the CSE group reported higher scores in comfort, overall well-being, and reduced nausea and anxiety. Moreover, our subgroup analysis revealed that the recovery-enhancing effect of CSE was particularly pronounced in the 45–64 age group, while no significant difference was observed in younger patients. This age-specific benefit may reflect diminished autonomic flexibility and functional reserve associated with middle age, rendering these patients more sensitive to the hemodynamic stability and superior analgesia provided by neuraxial anesthesia. In contrast, younger individuals may experience robust recovery regardless of anesthetic modality due to their greater physiological resilience [30].

The QoR-15 scores were classified into four levels to reflect different degrees of recovery severity [18]. The results indicated that significantly more patients were classified as having a “good” recovery and a smaller number as having a “moderate” recovery in the CSE group. This emphasizes the effectiveness of the CSE technique. Furthermore, the complete absence of patients experiencing “poor” recovery in the CSE group further highlights its positive impact on the quality of postoperative recovery.

Our findings indicate that opioid usage was reduced in the CSE (Combined Spinal-Epidural) group, which aligns with previous literature showing that neuraxial methods can lead to decreased opioid consumption and requirements [31, 32]. Opioids might lead to several side effects, such as PONV, sedation, itching, respiratory depression, and gastrointestinal motility disturbances [33]. The lower PONV incidence in the CSE group may be the result of reduced exposure to opioids [25]. Although we found no significant differences in the “physical comfort” subdomain, the specific QoR-15 item related to nausea and vomiting showed significant improvement in the CSE group.

Some studies suggest neuraxial anesthesia may lead to shorter mobilization times and reduced hospital stays [29, 34, 35]. However, the differences in the time to mobilization or the hospitalization length were not significant in the two groups. One possible explanation is that both groups received neuraxial procedures, such as epidural catheters, which minimized differences in these outcomes. Furthermore, local clinical protocols could have influenced the mobilization and discharge times, causing them to be longer than typically reported in the literature. Surgeon satisfaction was also similar in both groups. This might be because the choice of anesthesia was made collaboratively by the anesthesiologist and surgeon based on their preferences, which could introduce some selection bias.

This study has several limitations. Due to its prospective observational design, definitive causal relationships cannot be established, and the nonrandomized allocation of anesthesia types may introduce selection bias. Additionally, the single-center approach and limited sample size could restrict the generalizability of the findings. Since the QoR-15 scale is a subjective measure, it may be influenced by individual patient perceptions. Furthermore, the absence of long-term follow-up prevents any conclusions about medium and long-term outcomes. Finally, the use of tramadol as a rescue analgesic may be considered a limitation due to its relatively weaker analgesic efficacy and higher risk of postoperative nausea and vomiting (PONV). Although its selection was based on institutional protocols prioritizing respiratory safety, this choice may have impacted recovery-related patient-reported outcomes and may not represent the optimal approach in all clinical settings.

## Conclusion

This prospective observational study demonstrates that CSE anesthesia significantly enhances the recovery quality after the operation in those undergoing abdominal hysterectomy. Specifically, it provides effective early pain relief, reduces opioid consumption, and lowers nausea and vomiting, all of which contribute to improved patient comfort and emotional well-being. These findings suggest that CSE is valuable for developing optimal anesthetic strategies for surgical patients. Future large-scale, multicenter, randomized trials are necessary to validate and expand upon these observations, providing definitive guidance for clinical practice.

## Supplementary Information

Supplementary Material 1.

## Data Availability

The datasets generated and/or analyzed during the current study are not publicly available due to ethical restrictions related to the confidentiality of personal data, but are available from the corresponding author on reasonable request.

## Abbreviations

CSE: Combined Spinal-Epidural
GE: General Anesthesia with an Epidural Catheter
ASA: American Society of Anesthesiologists
PONV: Postoperative Nausea and Vomiting
NRS: Numerical Rating Scale
PCA: Patient Control Analgesia
QoR-15: Quality of Recovery-15
